# Patients' Perspectives on the Feasibility, Acceptability, and Impact of a Community Health Worker Program: A Qualitative Study

**DOI:** 10.1089/heq.2020.0159

**Published:** 2021-04-09

**Authors:** Wei Chang, May Oo, Adriana Rojas, April Joy Damian

**Affiliations:** ^1^ Weitzman Institute, Community Health Center, Inc., Middletown, Connecticut, USA.; ^2^ Department of Mental Health, Johns Hopkins University Bloomberg School of Public Health, Baltimore, Maryland, USA.

**Keywords:** community health worker, community health center, patient centeredness

## Abstract

**Purpose:** To examine patients' perceptions of the feasibility, acceptability, and impact of a safety net-based community health worker (CHW) program.

**Methods:** Semistructured interviews with patient participants diagnosed with type 2 diabetes (*n*=13) were analyzed using a traditional text analysis method based on grounded theory.

**Results:** This study highlights that the CHW program can improve satisfaction in accessing health services and community resources, and overall health outcomes of patients in safety net practices.

**Conclusion:** Patients' overall positive perception of the CHW program suggests that the intervention may be a viable solution to address the health and social needs of patients in safety net settings.

## Introduction

Community health workers (CHWs) are trusted members of the community with extensive knowledge about community resources and work as an integral liaison between community members and local services.^1^ Recent studies have found a wide range of positive outcomes associated with implementation of a CHW intervention program. Evidence from randomized clinical trials suggest CHW interventions led to improved health outcomes, better quality of care, and reduction in hospitalizations.^2–4^ Moreover, systematic reviews of CHW interventions suggest that CHW interventions can significantly reduce health care utilizations and cost as well as provide cost-effective interventions for certain health conditions, especially among underserved populations.^5,6^ However, despite a growing body of literature on how CHWs can improve health outcomes and decrease health care utilization, there is limited evaluations and knowledge around the effectiveness of CHW programs that is based on patients' perception. Thus, there is a need for research that examines the feasibility, acceptability, and impact of the pilot CHW program.

This study focuses on patients who have participated in the CHW pilot program to (1) explore patients' perspectives on the feasibility of the CHW program, including the timing and frequency, location, and attendance at sessions, (2) understand the acceptability of the CHW program, specifically with respect to the format and content of the intervention, and in comparison with other available supports, and (3) evaluate the impact of the CHW program, particularly in the areas of patients' knowledge of and access to medical and community resources, relationship with the health care team, as well as the program's impact on patients' social support, self-efficacy, and patient-stated goals.

## Methods

### Participants and procedure

Community Health Center, Inc. (CHCI) has adopted the outpatient Individualized Management of Patient-Centered Targets approach,^7^ which is a 6-month CHW intervention where patients work with a CHW to achieve their stated health goals. The intervention included 31 adults aged 18 years or older diagnosed with type 2 diabetes (with an glycosylated hemoglobin ≥8) and indicated English or Spanish as their primary language. All patients (*N*=31) who participated in the CHW pilot intervention were invited to participate in a study. Patients who called the research line to opt out were excluded. This study was approved by the CHCI Institutional Review Board. A subset of eligible participants (*n*=13) who previously enrolled in the CHW program provided consent to participate in the study. Semistructured interviews (Appendix A1) were conducted with consented patients to understand their experiences with the CHW program.

### Data analysis

Interview transcripts were analyzed using a traditional text analysis method based on classic grounded theory.^8^ The construction of traditional text analysis involves identifying themes from the interview transcripts, marking up the texts that relate to the themes of the research, eliminating the texts that are not related to the subject of the research, and sorting them into thematic categories.^8–10^ Two researchers first coded independently, followed by mutual discussions with the principal investigator to ensure the accuracy of coding.

## Results

### Patient demographics

Table 1 includes a summary of demographics of the sample (*n*=13). In the present sample, the average age of participants was 56 years (standard deviation=9.2). The majority of participants identified as female (69.2%) and Hispanic or Latino (69.2%).

**Table 1.** Participant Demographics (*n*=13)

Characteristics	Patients, *n* (%)
Gender	
Male	4 (30.8)
Female	9 (69.2)
Age	56 (±9.5)^a^
Race	
White	4 (30.8)
Unreported/refused to report	7 (53.9)
Other race	2 (15.4)
Ethnicity	
Hispanic or Latino	9 (69.2)
Not Hispanic or Latino	4 (30.8)

^a^ Mean±standard deviation.

### Outcomes related to the CHW program

#### Patients' perspectives on the feasibility of the CHW program

Participants talked about the feasibility of the CHW program, including timing of the sessions, frequency of interaction with CHW, location of meetings, and reasons behind missed session(s) (Table 2). The results suggest an overall satisfaction with the length of the program, flexible location arrangements, and flexibility in scheduling. All participants (*n*=13) reported that the timing and frequency of sessions were adequate. With regard to the location of the engagements, the majority (*n*=10) mentioned the clinic as a convenient location for them to meet. However, three participants preferred their home as the primary location for meetup given their health conditions. In addition, 11 out of 13 participants had missed meetings due to forgetfulness (*n*=1), work schedule (*n*=3), transportation issues, mobility issues, and health conditions (*n*=7).

**Table 2.** Outcomes Related to *Feasibility* of the Community Health Worker Program

Themes	Participant quotation
Timing and frequency of sessions	“Length of the program, like that interaction so would like it to be longer.” *02*
	“100! She always made sure that it was convenient for me. It wasn't about her or her schedule. She always made sure it was good timing. I met with her as much as I needed. Maybe once a week, but if I called her and needed her we met more.” 06
	“Even when we are not meeting in person, I know I can still call her and connect by phone should I need assistance with anything.” 11
Community venues vs. clinical settings	“Yes, it's fine for me to meet her at the clinic. Whenever I have trouble meeting her there, she's willing to make other arrangements so we can meet.” *07*
	“Sometimes we met in person at the clinic, and sometimes at home. Sometimes she would come to my place since it was convenient. Also, when I'm not able to go to the clinic, she comes to my place since she knows my breathing and mobility issues. I would prefer to meet with the CHW more often at my place my health conditions since it's sometimes difficult for us to meet in person at the clinic.” 09
Attendance at sessions	
Forgetfulness	“Sometimes he forgets he has an appointment with her. Sometimes he missed session because he forgot, having a piece of paper and call the day of the appointment is helpful.” *01*
Work schedule	“Yes, sometimes I've had to miss sessions due to last-minute needs or emergencies at work.” *11*
Transportation issues, mobility issues, and health conditions	“Sometimes I wasn't able to meet her at the clinic because I didn't have transportation or because I had trouble with my mobility since I've had an amputation due to my diabetes.” *09*
	“Sometimes I'm not feeling well, sometimes I feel lightheaded or have a headache related to my diabetes, so I have cancel my session with her.” 12
	“Yes, but in these instances, I would call her in advance and reschedule. Also, if I couldn't physically come to the clinic because I've been very sick, my arthritis makes it very difficult to get around, even getting out of bed is difficult, and causes a lot of pain. She's willing to meet me at home though so we are able to get together.” 13

#### Patients' perspectives on the acceptability of the CHW program

Participants shared perspectives on the acceptability of the CHW program, specifically on the content, perception of the CHW, in comparison with other available nonmedical supports, and key recommendations for improving the program (Table 3). All participants (*n*=13) reported being satisfied with the program, including the usefulness and appropriateness of topics covered, as well as the structure and flow of the program. Moreover, all respondents (*n*=13) were satisfied with the CHW's professionalism, resourcefulness, knowledge, and engagement. When comparing the CHW program with other nonmedical supports, nine out of 13 participants mentioned they could not compare since they have not received any other supports (*n*=6) or the focus areas of other programs and interventions were different (*n*=3). In terms of recommendations for improving the program, five participants suggested the expansion of the program, which reflects the need to reach more people who could benefit from the CHW program by gaining access to the required medical and social support.

**Table 3.** Outcomes Related to *Acceptability* of the Community Health Worker Program

Themes	Participant quotation
Content	“Leonora is the best thing that ever happened to me. When I first met her (oh my god I'm going to cry). When I met with her I was at the lowest point in my life. I am usually the one to help people and I was embarrassed that I needed help. She told me that there's nothing to be embarrassed about and that I'm here to help you.” *06*
	“I know I can always call her for whatever I need, for example, if I need help getting to my medical appointments, or filling out paperwork related to social security, especially since English is not my native language so I have challenges understanding what they are asking me in the paperwork and how to complete it.” 07
	“The program has helped me a lot because she understands me very well, she's helped me from everything from managing my diabetes, becoming more physically active, and just feeling better about myself.” 11
	“For me, it's helped me tremendously because I've been worried and overwhelmed by my health. I get help from everything from scheduling and rescheduling my health appointments, keeping track of my medications and taking them correctly, and just understanding how to be healthy.” 13
Perception of CHWs	“Positive experience with CHW, if she doesn't know the answer she'll look for the answer and will get back to her within a day or two, very professional.” *02*
	“I have told several family members that they should go see Leonora because from day one she has been keeping track of everything and helping me get what I need. I'm very satisfied. She's amazing at what she does. I didn't think in the beginning that it was going to be a good thing. I thought it was just another appointment but now I look forward to it.” 04
	“Leonora as a person, she makes the program. Because without a compassionate person like her the program wouldn't work. I miss talking to her and having someone I can unload to without feeling judged. She doesn't judge no matter what. These days lots of people judge you. The only other person like Leonora is my mother.” 06
Contrast with other nonmedical supports offered	“I don't think there's any comparison since she helps me from everything from understanding my medical issues to having access to transportation.” *08*
	“I'm in another program called Silver Sneakers, but I really can't compare the two. Silver Sneakers focuses on physical activity like Zumba, chair exercises, but the challenge is that I have to go to the YMCA to participate in this program. I don't always have the transportation to get there, nor do I feel comfortable walking by myself given my physical condition. It's more convenient to meet at my place with the CHW program.” 12
Key recommendations for improving the program	“I think more people need access to this type of program since it's very personal and holistic.” *08*
	“I would recommend expanding the program so that she's not the only person in this role since there's such a need for patients who have trouble getting to their doctor's appointments, challenges with understanding what the doctor is saying or the doctor's instructions especially since English is not my native language. Also, it's not just the medical needs, but I know I can approach with whatever other needs I might have.” 10
	“The challenge is that she (the CHW) is only one person and she's having to deal with all of us patients who have such complex and unique needs. I need to go to the pharmacy, get groceries, go to my doctor's appointments, but I know she has other patients to also help, so if she had more support, even a shuttle to get us around so she doesn't have to always drive us around, that would help. I hope they keep this program going since it really is a huge benefit to patients, and that the organization makes an effort to support her (CHW) since it is a lot of work.” 12
	“I think the program needs to expand since other patients need people like her who are patient and can help navigate the health system since it can be very overwhelming and complex without this kind of support that she provides.” 13

CHWs, community health workers.

#### Patients' perspectives on the impact of the CHW program

When describing their perception regarding the impact of the CHW program, participants focused on three major themes: health care access and engagement, health-related social needs and resources, and self-efficacy and patient goals (Table 4). Specifically, patients noted the positive impact of the CHW program on improving medical knowledge, access to medical services, and self-efficacy. Participants stated their understanding of the importance of diabetes management and self-care (*n*=10), medication adherence (*n*=3), and keeping medical appointments (*n*=2) have improved as a result of participating in the program, and contributed to the adoption of healthy behaviors. Moreover, participants shared that issues around access to medical services such as appointment scheduling (*n*=7), medication retrieval (*n*=2), and transportation (*n*=6) have shown improvement with the integration of the CHW program to usual care. Furthermore, related to self-efficacy, participants demonstrated the support of CHW helped reduce anxiety (*n*=3), boost self-esteem (*n*=8), and improve the overall patient experience and health outcome (*n*=8).

**Table 4.** Outcomes Related to *Impact* of the Community Health Worker Program

Themes	Participant quotation
Impact on health care access and engagement	
Impact on medical knowledge	“When she first explained to me that everybody that I see can help me feel better it started to make more sense. She's made me take more time for my health. I work a lot of hours and didn't use to keep appointments. She made me understand why I need to keep my appointments.” *04*
	“I have a better understanding of how to test my blood sugar, how to manage my diabetes, navigate my different medical appointments, and also engage in more physical activity.” 08
	“I have anxiety, which gets worse when I'm trying to exercise since I get nervous about my condition. However, being with her I know I can stay calm and exercise. I also know how to better manage my diabetes, like understanding how to take my own blood sugar.” 12
Impact on access to medical services	“Before, I had challenges with transportation, which made it difficult to always make it to my medical appointments and get around. But, she (CHW) has been so helpful in making sure this is addressed. She's helped me retrieve my medications. She has helped me navigate the referrals I have to other providers since I don't always know how or who I'm seeing and when, so it's helpful to have her organize all my medical appointments.” *07*
	“She calls me with reminders, and it's great that we have great communication with each other. She serves as my translator to help me navigate the health system. For example, sometimes I need help changing my medical appointments or communicating with my health team; she's there to assist me with all of these needs. In addition to my diabetes and bronchitis, I also have arthritis and hypothyroidism, and other health issues. She's helped me navigate the different medical appointments that I have for these conditions, which has been so helpful.” 09
	“Given my arthritis, she has been so helpful in making sure my physical therapy location is easy for me to get to, and that I'm able to get there with ease. When I needed transportation to get to New Haven for the leg I had operated, she was helpful in finding a way for me to get there.” 13
Impact on engagement with health care team	“Reached out to PCP and nurse before working with CHW so that has not changed much. Didn't change relationship with PCP or nurse.” *01*
	“My doctors are doing more stuff and tests on my eyes and things. My doctor never suggested them before, I know that for a fact.” 03
Impact on health-related social needs and resources	
Impact on access to health-related social needs	“Food stamps, sometimes CHW gives him the bus tickets, sent them by mail. Before joining the CHW program, appointment (substance use clinic) on Wednesdays in Rushford and cannot get back (daughter or sister would drop him off). Now CHW has gotten a bus pass for him and he has a ride back.” *01*
Impact on knowledge of community resources	“Resources—there aren't many, which I've always known since I worked for shelters. Leonora tried to find new resources, but it's hard. She tried to find programs to help me, but there weren't none.” *06*
	“She's informed me about resources in the community, she's asked me if I need anything in the community, and I've said I don't need them and it's better off for someone who needs them more to have them, but it's still nice of her to ask. She's informed me about resources to help pay for utilities, for example, but I don't really need it. I think someone who is in more need should use these services, but I'm still grateful.” 11
	“I haven't really needed help with this since I already am enrolled in multiple benefits including Medicare/Medicaid and SNAP.” 13
Impact on social support	“I've always had my mom and my kids. I haven't had many friends because they are always negative. Leonora helped me get comfortable opening up. I talk to my daughter better now and not just argue. I used to worry, worry, worry. Now I give something everything I have and then let it go.” *06*
	“I live alone so sometimes I feel isolated. However, now that I'm in this program, I feel more connected, especially since I'm able to connect my care team on a more regular basis for my diabetes.” 08
	“I haven't really had any challenges with my social support system, so I can't say she's really helped me in this capacity.” 10
Impact on self-efficacy and patient goals	
Impact on self-efficacy	“She had very low self-esteem before. CHW said every woman has something beautiful inside them, stuck in her mind.” *02*
	“She helped me get the confidence to work two jobs to do what I needed to do. She gave me the confidence to help me sell myself and get a job. One thing she always told me is ‘you got this, don't give up.’ If I feel like everyone is against me, I remember those words and keep going.” 06
	“I know I have someone who is willing to help me so I'm not on my own in getting what I need, I know I have someone who is advocating for me in whatever situation I find myself in and I can have faith and confidence that the situation will work in my favor.” 13
Impact on patient stated goals	“Oh, it's helped me a lot because first of all my diabetes was out of control and now my diabetes is in a non-diabetic state. She's on top of me! it's good, it's okay. My doctor is surprised because when I first started going there my A1C was at a 13 and now it's a 5.” *04*
	“We worked on housing and self-esteem and getting work. She also helped me with my faith and patience and knowing that where I was at that point in my life was okay and that I needed to keep going and keep fighting. I was homeless and sick when we met. My son had just gone to jail. I didn't think things would get better, but she didn't let me give up. We worked on me getting a job. I'm working almost full time now.” 06
	“My goal in the beginning was to lose weight, this was the first thing I shared with her. My weight is still fluctuating, sometimes I lose weight, then I gain it back because it's hard for me to get to the YMCA.” 09

### Patient engagement in the CHW program

Participation in the CHW program indicated the evidence of the effectiveness on health outcome (Table 5). The majority of patients (69.2%) receiving CHW intervention demonstrated promising improvement in their A_1c_ level, whereas the remainder of the participants showed unchanged (15.4%), and increased (15.4%) A_1c_ level. Furthermore, the attendance of the program has a positive impact on patient engagement, allowing patients to meet their stated health goals. The results displayed that the best-performing participants (61.5%) were the ones who were present during almost all meetings with CHW. Patients who were contacted over the phone due to their medical conditions or other personal issues reported no significant improvement in their A_1c_ level regardless of their good attendance.

**Table 5.** Patient Engagement in the Community Health Worker Program (*n*=13)

Changes in participants' HbA_1c_ level and number of missed meetings			
Participant ID	A_1c_ at baseline	A_1c_ at postintervention	No. of missed meetings
1	12.4	6.7	0
2	9.8	9.9	6
3	8.1	7.8	2
4	7.0	6.0	0
5	10.4	7.7	0
6	7.7	6.6	2
7	11.2	10.6	4
8	6.4	5.8	6
9	11.6	7.8	0
10	6.9	6.9	4
11	14.0	14.0	1^a^
12	8.4	9.3	6
13	8.0	7.9	0^a^
Characteristics of participants' engagement			
		Patients, *n* (%)	
HbA_1c_ level			
Decreased A_1c_ level		9 (69.2)	
Unchanged A_1c_ level		2 (15.4)	
Increased A_1c_ level		2 (15.4)	
No. of missed meetings			
0–2		8 (61.5)	
3–5		2 (15.4)	
>6		3 (23.1)	

^a^ Patients were contacted over the phone due to medical conditions or other personal problems.

HbA_1c_, glycosylated hemoglobin.

## Discussion

This study offers qualitative insights into patients' perspectives on the feasibility, acceptability, and impact of a CHW program. A CHW program is one-of-a-kind intervention to improve patient's self-efficacy or medication adherence,^11^ and to increase access to the hard-to-reach population.^12^ This study suggests the effectiveness of the CHW program in the areas of increasing health knowledge and improving access to needed health services and health outcomes, which aligns with previous research.^13–16^

In addition, the results of this study demonstrate the positive effect of professionalism of the CHW in enhancing patient self-efficacy and trust. With the help of the CHW, patients acknowledged that they built up skills and confidence to achieve their target goals by modifying their lifestyle behaviors, which is consistent with earlier research that documented the benevolent relationship between CHW and community.^17–19^ By understanding patients' experiences with the CHW program, the findings advocate for improvements and expansion of the CHW program at CHCI.

This study has several limitations. First, social desirability bias might be an issue since one of the data collectors has exposure to patients. This may have reported a higher patients' perception of and satisfaction with the CHW program. Comparing patients' stated goals in the semistructured interview with their baseline goals might mitigate this bias. Second, a small sample size where the majority of the participants identified as female, unreported race, and Hispanic or Latino might affect the generalizability of this study. Nonetheless, 6–12 interviews were sufficient enough to achieve thematic saturation.^20^

## Conclusion

With these limitations in mind, the findings from this study have several implications on policy, practice, and research. First, the study highlights how integration of CHWs into clinical care teams could encourage patient self-advocacy by providing culturally appropriate health education, interpreting services, and health care system navigation. Establishing appropriate funding mechanisms such as grants, incentives, and reimbursement would allow for the sustainability and scalability of CHW programs within safety net settings. Similarly, health care practices working to address disparities and promote patient-centered care should invest in CHWs as extensions of the care team and the role of CHW needs to be recognized more for their contribution to increase health access and improve health outcomes. Further research regarding the solution to common pitfalls of the CHW program can help improve patient self-advocacy by addressing their medical and social needs, and health outcomes by strengthening the capacity of the CHW.

## Abbreviations Used

CHCI: Community Health Center, Inc.
CHW: community health worker

## Appendix

### Rationale

The purpose of the semistructured interviews is to understand, from the patients' perspective, the (1) feasibility, (2) acceptability, and (3) impact of the Community Health Center Community Health Worker Program piloted at the Meriden clinical site. Interviews with patients currently enrolled in the community health worker (CHW) pilot program (*N*=31) will be conducted at the conclusion of their engagement in the 6-month intervention. The research team will then triangulate data from the semistructured interview described here with data collected at baseline from the “Meet the Patient” assessments, to draw conclusions about the CHW pilot program. To our knowledge, this is the first evaluation of a U.S.-based CHW program grounded in the experiences of patients.

[Insert introductory prompt/consent]

### Feasibility

#### Timing and frequency of sessions

How satisfied are you with the timing of your sessions with the CHW? The number of times you met with the CHW?

Probe about patient's satisfaction with the timing when the sessions were conducted, including (in)convenience. Probe about how often the patient met with the CHW, and if the frequency was too little/much, adequate.

#### Community venues versus clinical settings

How satisfied are you with where you and the CHW would decide meet?

*Probe about patient's satisfaction with the location of engagements (*e.g., *clinic, home) as well as modes of interaction (*e.g., *in person, phone).*

#### Attendance at sessions

Was there ever a time you had a meeting scheduled with the CHW and were unable to come?

Probe about reasons why patient missed session(s) with CHW.

### Acceptability

#### Content

Please describe your overall experience with the program.

Probe about patient's overall satisfaction with the program, including usefulness and appropriateness of topics covered, as well as structure and flow of the program.

#### Perceptions of the CHW

Please describe your overall experience with the CHW.

Probe about patient's overall satisfaction with the CHW, including professionalism, resourcefulness, knowledge, and engagement.

#### Contrast with other nonmedical supports offered

How would you compare your experience in the CHW program with other types of supports offered?

*Probe about how patient's participation in CHW program compared with other similar programs, including what worked well in CHW program compared with other programs and* vice versa.

#### Key recommendations for improving the program

What are your recommendations for improving the program?

*Probe about what changes the patient would recommend implementing (*e.g., *length, structure, and content of program).*

### Impact

#### Impact on knowledge (medical)

To what extent, if at all, has your understanding of your medical care improved as a result of participating in this program?

Probe about changes in patient's understanding of health needs, medical care plan, medications taken/side effects (medication adherence), test results, referrals to specialists, discharge instructions if recently hospitalized.

#### Impact on knowledge (community resources)

To what extent, if at all, has your understanding of resources available in the community available to you improved as a result of participating in this program?

*Probe about changes in patient's understanding of community resources (*e.g., *transportation, childcare, insurance, debt collection, drug and alcohol counseling, food assistance, and utilities).*

#### Impact on access to medical services

To what extent, if at all, has your access to medical services improved as a result of participating in this program?

*Probe about changes in patient's ability to access needed medical services. Probe about barriers that may have existed before engagement with CHW (*e.g., *transportation challenges, not understanding care plan), and to what extent these challenges have been addressed through this program.*

#### Impact on engagement with health care team

To what extent, if at all, has your engagement with the health care team improved as a result of participating in this program?

Probe about changes in patient's engagement with the health care team, including the patient's perception of the Community Health Worker being part of the health care team.

#### Impact on access to health-related social needs

To what extent, if at all, has your access to health-related social needs improved as a result of participating in this program?

Probe about changes in patient's capacity to access resources that support well-being (see examples of community resources listed earlier).

#### Impact on social support

To what extent, if at all, has your social support improved as a result of participating in this program?

*Probe about changes in patient's perception of support from others. Probe about particular types of support systems (*e.g., *family, friends, community, care team, and CHW).*

#### Impact on self-efficacy

How has participating in this program impacted your belief in your ability to succeed in specific situations/accomplish a task?

Probe about changes in confidence in ability to set goals and achieve them.

#### Impact on short-term goals

Please describe the progress you have made toward the short-term goals you identified at the beginning of the program.

Probe about specific short-term goals developed at baseline and steps taken to achieve goals. Probe about what supported progress toward short-term goals. Probe about challenges encountered in achieving short-term goals, and what would have been helpful to have in place to overcome identified challenges.

#### Impact on long-term goals

Please describe the progress you have made toward the long-term goals you identified at the beginning of the program.

Probe about specific long-term goals developed at baseline and steps taken to achieve goals. Probe about what supported progress toward long-term goals. Probe about challenges encountered in achieving long-term goals, and what would have been helpful to have in place to overcome identified challenges.

### Closing

Is there anything else you would like to share that I have not already asked?

Thank participant for his/her time.
